# Insidious presentation of intussusception with appendicitis

**DOI:** 10.1093/jscr/rjaa578

**Published:** 2021-01-18

**Authors:** Nargus Ebrahimi, Yu-Ting Yeh

**Affiliations:** General Surgery, Blacktown Hospital, Blacktown, Australia; General Surgery, Blacktown Hospital, Blacktown, Australia

## Abstract

Intussusception in relation to appendicitis is an uncommon occurrence and is rarely described in the literature. We describe a case of diagnostic uncertainty and finding of ileocolic intussusception associated with appendicitis in a 22-year-old male. The patient presented with a history of acute right-sided abdominal pain. He underwent a computed tomography scan showing ileocolic intussusception following an operation with the finding of an inflamed appendix, which was likely to have served as a mechanical lead point of the intussusception. Due to the presence of ischaemia of the right colon, he underwent a right hemicolectomy.

## INTRODUCTION

Intussusception is a well-described phenomenon; however, it is rare in adults. Only 5% of cases occur in the adult population, which represents 1–5% causes of bowel obstruction in adults. The majority of cases affect children, with a mean age between 6 and 18 months [1]. Intussusception involves the invagination of a proximal segment of bowel, called the intussusceptum, into a distal segment of bowel, called the intussuscipiens. Various parts of the bowel may be involved. We describe a case of appendiceal–ileocolic intussusception.

## CASE REPORT

A previously well 22-year-old male presented to the emergency department with a 6 h history of abdominal pain, associated with nausea, vomiting and subjective fevers. He had sudden onset of epigastric pain after drinking a smoothie, sharp and intermittent in nature. He reported a similar self-resolving episode of pain about a year ago. On examination, he had a temperature of up to 37.9 and was haemodynamically stable (heart rate 82, blood pressure 116/78, respiratory rate 16 and saturations 99% on room air). His abdomen was soft with mild tenderness noted in the right upper quadrant and right iliac fossa.

Blood tests on admission demonstrated a white cell count of 3.7 and elevated C-reactive protein (CRP) of 83. A venous blood gas showed a blood lactate of 0.9. The chest X-ray was unremarkable.

**Figure 1:**
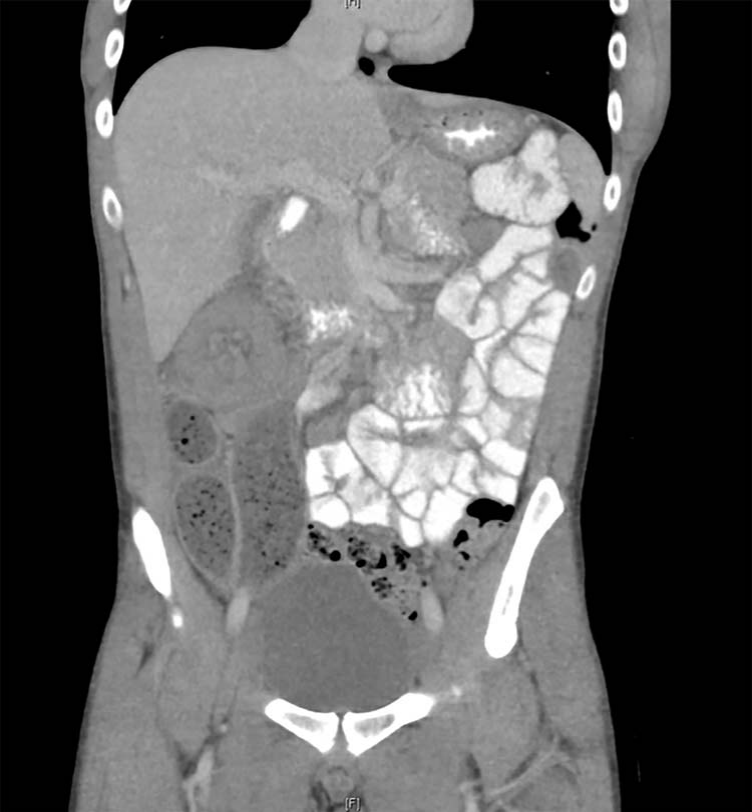
CT of the abdomen and pelvis with coronal view showing ileo-colic intussusception.

**Figure 2:**
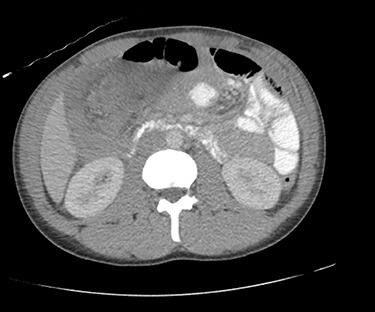
CT of the abdomen and pelvis with axial view showing ileo-colic intussusception.

Due to diagnostic uncertainty, the patient underwent a computed tomography (CT) of the abdomen and pelvis with intravenous and oral contrast, which demonstrated ileo-colic intussusception, a distended ileum and no other features of bowel obstruction. Coronal and axial views of the CT scan showing the ileocolic intussusception are displayed in Figs 1 and 2, respectively.

The patient was taken to theatre that evening. During the laparoscopy, the ileocolic intussusception was manually reduced, revealing a thickened inflamed appendix, which had invaginated within the right colon. The involved bowel appeared haemorrhagic and due to the signs of bowel necrosis, a decision was made to proceed with a right hemicolectomy.

Histopathological examination of the resected specimen revealed acute inflammation and oedema of the colon. The appendix was acutely inflamed with features of fibrosis.

## DISCUSSION

Intussusception in conjunction with appendicitis is uncommonly reported in the literature. In our patient’s case, the appendiceal inflammation observed macroscopically and microscopically could have been the primary event, serving as a lead point for the intussusception. Alternatively, appendiceal inflammation could have been secondary to bowel strangulation or ischaemia caused by the intussusception. This ‘chicken or egg’ scenario has been similarly described elsewhere [2, 3]. Intussusception can be defined based on the location in the bowel, with types including appendiceal, appendiceal–ileocolic, ileocolic, colocolic and enteroenteric [1]. The location is usually at the junction between mobile and fixed bowel. In our patient’s case, the intussusception could be described as the ileocolic or appendiceal–ileocolic type.

In children, most cases are idiopathic, of the ileocolic type, and treatment is often with an air or contrast enema, successful in 80% of cases [1, 4]. In contrast, adult intussusception has an identifiable cause (i.e. a mechanical lead point) in 90% of cases, with the remainder being idiopathic. Neoplastic causes serve as the lead point in 67% of cases, half of which are malignant caused by adenocarcinoma, metastatic disease or lymphoma, whereas the other half are benign such as lipoma and adenomatous polyps. Non-neoplastic causes include adhesions, Meckel’s diverticulum, and infections such as tuberculosis, Crohn’s disease and granulomas [1, 5].

Treatment of intussusception in adults is distinct from the approach in the paediatric population, and this discrepancy traditionally relates to the difference in the cause of intussusception. Owing to the large number of cases caused by malignancy, intraoperative reduction is not recommended and segmental resection is advocated [6]. In addition, distinct to colonic intussusception, there is a growing acknowledgement that cases of small bowel intussusception can be managed non-operatively with spontaneous resolution [1].

Adult intussusception does not present with the classical triad of severe abdominal pain, bloody diarrhoea and palpable mass recognized in infants. Adults may exhibit non-specific symptoms including abdominal pain, distension, nausea, vomiting, per rectal (PR) bleeding and <9% have a palpable mass [5]. CT features include presence of an entering wall, a returning wall, intervening mesenteric vessels, fat and intraluminal space. In the presence of a lead point, there may be a target like mass and features of proximal obstruction [7].

Our case highlights the diagnostic difficulty associated with intussusception, in particular, with a young patient who appeared well and did not have leukocytosis. The presence of persistent tenderness on examination and an elevated CRP warranted further evaluation by CT, leading to a preoperative diagnosis of intussusception. Furthermore, intraoperative evaluation in our patient’s case led to a diagnosis of ileocolic intussusception with appendicitis as the primary or secondary event, with no other lead point identified. This case highlights the need for a high index of suspicion in well-appearing young adults who present with abdominal pain without a diagnosis.
